# Supplementary material for: Conventional and hyperspectral time-series imaging of maize lines widely used in field trials
Source: Gigascience. 2017 Nov 24;7(2):1–11. doi: 10.1093/gigascience/gix117 (PMC5795349; doi:10.1093/gigascience/gix117)
Supplement: GIGA-D-17-00187_Revision-1.pdf [file gix117_giga-d-17-00187_revision-1.pdf]

GigaScience

Conventional and hyperspectral time-series imaging of maize lines widely used in field trials

--Manuscript Draft--

Manuscript Number:	GIGA-D-17-00187R1
Full Title:	Conventional and hyperspectral time-series imaging of maize lines widely used in field trials
Article Type:	Data Note
Funding Information:	Nebraska Corn Board (88-R-1617-03) — Dr. James C Schnable<br>Iowa Corn Board — Dr. James C Schnable<br>National Science Foundation (OIA-1557417) — Dr. James C Schnable
Abstract:	Background: Maize (Zea mays ssp. mays) is one of three crops, along with rice and wheat, responsible for more than 1/2 of all calories consumed around the world. Increasing the yield and stress tolerance of these crops is essential to meet the growing need for food. The cost and speed of plant phenotyping is currently the largest constraint on plant breeding efforts. Datasets linking new types of high throughput phenotyping data collected from plants to the performance of the same genotypes under agronomic conditions across a wide range of environments are essential for developing new statistical approaches and computer vision based tools.<br><br>Findings: A set of maize inbreds - primarily recently off patent lines -- were phenotyped using a high throughput platform at University of Nebraska-Lincoln. These lines have been previously subjected to high density genotyping, and scored for a core set of 13 phenotypes in field trials across 13 North American states in two years by the Genomes to Fields consortium. A total of 485 GB of image data including RGB, hyperspectral, fluorescence and thermal infrared photos has been released.<br><br>Conclusions: Correlations between image-based measurements and manual measurements demonstrated the feasibility of quantifying variation in plant architecture using image data. However, naive approaches to measuring traits such as biomass can introduce nonrandom measurement errors confounded with genotype variation. Analysis of hyperspectral image data demonstrated unique signatures from stem tissue. Integrating heritable phenotypes from high-throughput phenotyping data with field data from different environments can reveal previously unknown factors influencing yield plasticity.
Corresponding Author:	James Schnable<br>University of Nebraska-Lincoln<br>Lincoln, NE UNITED STATES
Corresponding Author Secondary Information:	
Corresponding Author's Institution:	University of Nebraska-Lincoln
Corresponding Author's Secondary Institution:	
First Author:	Zhikai Liang
First Author Secondary Information:	
Order of Authors:	Zhikai Liang<br>Piyush Pandey<br>Vincent Stoerger<br>Yuhang Xu<br>Yumou Qiu<br>Yufeng Ge

Powered by Editorial Manager® and ProduXion Manager® from Aries Systems Corporation

	James C Schnable
**Order of Authors Secondary Information:**	
**Response to Reviewers:**	We would like to thank both the editorial staff of Gigascience and both peer reviewers for taking the time to evaluate the previous draft of this manuscript and provide extremely helpful feedback. It is our belief that this revised manuscript is significantly improved as a result of the changes suggested in the previous round of review.<br><br>Reviewer #1:<br><br>In the study entitled "Conventional and hyperspectral time-series imaging of maize lines widely used in field trials", Liang et al. performed high-throughput phenotyping (HTP) in a set of maize inbreds. The dataset is especially valuable through integrative analysis with matched field data from different environments and genotypic data.<br><br>Major points:<br>1. It's unclear what automated phenotyping system was used in the study. Is it commercial and available to other users?<br><br>This was a serious omission. We have added information on this to the manuscript. This is the first complete dataset to be released from the UNL greenhouse innovation center phenotyping system. The UNL-GIC is based on an Lemnatec/Bosch style design and is available to both internal UNL researchers and external academic and private sector clients. We have added text to the methods and introduction sections to provide the same information summarized in this response.<br><br>2. One of the advantages of HTP is to enable us to extract a long list of phenotypic traits for the same plants from image data. However, in the study, the authors only showed some well investigated traits like plant height and projected area. It would be nice to indicate how many potential traits can be extracted from their image data, and how about the potential<br><br>We thank the reviewer for this valuable suggestion. We have added an additional concluding paragraph summarizing traits which have been extracted from different image types in the past and discuss other potential phenotypes which it may be possible to extract from these types of images in the future.<br><br>3. The authors provided infuorescence and thermal infrared data in the study. But these data were not shown in their analysis, correct? If not, it would be to have an evaluation of these data.<br><br>The reviewer is correct that the fluorescence and thermal infrared image data was included in the release but not analyzed in the short results section. Based on the concerns of both reviewer #1 and reviewer #2, in this revised version we include analysis and validation of the fluorescence imaging data -- extracting average fluorescence intensity measurements from plant pixels and testing the heritability of these values -- in results and in figure 4B and the additional code used to perform these analysis has been added to the associated github repository. We have also added discussion and references to previous literature demonstrating the utility of measurements of fluorescence intensity and its correlation to other plant traits such as chlorophyll content and abiotic stress responses.<br><br>4. In figure 2A and 2B, plant height showed higher correlation with manual data but biomass not. This is true because biomass is a complex phenotypic that cannot solely explained by a single measurements (plant project area). I have three related questions here.<br><br>1) which project area used for the analysis? PA0 or PA90?<br><br>We apologize for the ambiguity. We actually used the sum the the estimated number of plant pixels from the two offset side views. The manuscript has been revised to explicitly state this point at the place where we first introduce biomass estimation.

Powered by Editorial Manager® and ProduXion Manager® from Aries Systems Corporation

2) the correlation coefficient (r=0.72) is lower than existing results in maize (e.g., Muraya et al, 10.1111/tpj.13390). Is this possible due to the software used to derive the area measurement? If I correctly read the manuscript, the author used their in-house image processing pipeline. Did the author compare their results to the results generated by some published image-processing software?

We thank the reviewer for this suggestion. We have reestimated plant biomass using PlantCV2 (Noah et al. 2015 DOI: 10.1016/j.molp.2015.06.005; Gehan MA et al. DOI: 10.7287/peerj.preprints.3225v1). The correlation between the sum of plant pixel areas in the two side views and ground truth biomass measurements was (r=0.91). The large increase in accuracy in the PlantCV2 based analysis lead us to reevaluate the approach used in our own scripts, and identify a significant coding flaw which lead to incorrect plant pixel count data being using in our own calculations. We have revised figures 2B, 2C and 4B to use the corrected data, and provide a comparison of biomass estimates from the code used here, PlantCV2's code, and ground truth measurements in the revised manuscript.

3) Given the biomass is not well predicted by a single image-derived parameters, I would suggest to predict it by using multiple parameters (e.g., doi.org/10.1101/046656).

We agree with the reviewer that pixel counts should not have a perfect linear relationship with plant biomass. Factors such as the ratio of stems to leaves, and stem density and diameter vary significantly among genotypes and can produce significant differences in plant pixel counts from plants with equivalent quantities of biomass. We included the analysis of the relationship between plant pixel number and estimated biomass as single variable linear models of the relationship between plant pixel count and biomass are widely used in the literature (Noah F et al. DOI:10.1016/j.molp.2015.06.005; Golzarian MR et al. DOI:10.1186/1746-4811-7-2; Malachy T. Campbell MT et al. DOI: 10.1104/pp.15.00450; Ge Y et al. DOI: 10.1016/j.compag.2016.07.028), and to illustrate there that there is systematic genetically-linked bias in estimates of plant biomass generated using this method.

However, based on this suggestion by the review we now include the results from a second set biomass estimates generated using the four approaches proposed in the manuscript referenced above (multivariate linear model, MARS, Random Forest and SVM).* These models increased the accuracy of plant biomass estimates from r = 0.91 (single variable linear model) to r = 0.96 (the machine learning methods MARS). However, we show than even when using machine learning methods trained to estimate biomass from multiple traits extracted from multiple image types error in biomass estimate remains non-random and systematically biased towards over or under estimation of the biomass of specific genotypes. This information is now included in the main text.

5. Since the plants were randomly set on the pot, in principle there is no difference for the measurement of PA0 and PA90. But they are significantly different (figure 5)?

We appreciate the reviewer raising this point. On the first day of imaging or when plants reach to the two leaf stage, plants were arranged so that the major axis of leaf phylotaxy was parallel to the camera in the PA0 orientation and perpendicular to the camera in the PA90 angle. We have added this information to the manuscript in both the declarations and the legend of figure 4 (where the terms PA0 and PA90 are first introduced). As we feel that this point was a distraction from the central point of the manuscript, we have removed Figure 5 in this revised version of the manuscript.

Reviewer #2: This manuscript describes the generation of a time-series dataset of conventional and hyperspectral images of commonly known and important maize lines. The authors describe the methods of data collection and how it is useful, especially in conjunction with other already available datasets for the same lines. The authors begin to analyze the dataset generated, focusing on biomass measures and determining heritability. The authors conclude that they believe it is important and necessary to combine controlled environment data with field data to tackle problems facing crop production. I do have several comments about the manuscript in its current form:

1.My main concern about the manuscript is the amount of data use in the article. The manuscript was submitted as a 'Data Note', but it is not obvious this data is exceptional, rare, or novel as it was collected nearly 2 years ago.

The reviewer raises two distinct points here. The first is that this dataset may not be exceptional or rare. The second is that this dataset is almost two years old. Of the various points raised by reviewer #2, these concerns appears to be the most critical barriers to publication, so we have responded to them at some length.

Exceptionality of the dataset.

I think the reason the two of us are in disagreement about the exceptionality of this dataset is that we're comparing this dataset to different baselines. I believe that compared to published plant image datasets, our dataset is exceptional for two reasons. 1) The link between publically available field based data in a wide range of well characterized environments. 2) Hyperspectral imagery collected under controlled light conditions from individual corn (maize) plants.

Now at the same time, because Gigascience practices open peer review, I can see that the reviewer is part of a research group which has done, and continues to do groundbreaking work in both the image collection and phenotypic data extraction portions of plant phenotyping. It wouldn't surprise me at all if, compared to the internal datasets he and his collaborators have access to, the dataset we are trying to release to the community is rather small and not particularly novel or exciting.

The disconnect between these two assessments is a result of the second point that the reviewer raises.

Age of the dataset.

It has been my observation that the cycle time from data collection to publication in plant phenomics is significantly longer than in other scientific fields in which I have experience (particularly plant comparative genomics where it is often possible to go from idea to analysis to submitted paper in as little as six months). I don't want to base my position on a qualitative gut feeling, so I dug into the literate a bit to see if that gut feeling is supported by quantitative data. In many cases manuscripts do not report the date of phenotypic data collection -- which is whole concern of its own but beside the point today -- but in the cases where dates of data collection were reported, the lag time was quite significant.

Chen et al Dec 2014 10.1105/tpc.114.129601 used data collected in July 2011 (3.5 years)
Campbell et al Aug 2015 10.1104/pp.15.00450 used data collected in 2013 (2.5 years)
Muraya et al Feb 2017 10.1111/tpj.13390 used data collected in 2011-2012 (5+ years)
Zhang et al Mar 2017 10.1104/pp.16.01516 used data collected in 2011-2012 (5+ years)
Feldman et al June 2017 10.1371/journal.pgen.1006841 used data collected in 2013-2014 (3+ years)

I suspect that part of the delay between data collection and publication for plant phenomic datasets is the fact that computational and statistical methods for the analysis of high throughput plant phenotyping data remain much less mature than those for other data types encountered by plants scientists. Right now it is very hard to interest third party computer scientists and statisticians without pre-existing collaborations with plant scientists in developing new methods for plant phenomics given the dearth of well documented and freely usable datasets. It was and is our hope that this dataset and others like it can help to bring more such researchers into the community.

Summary

In conclusion, while I agree with the reviewer that the field of plant phenomics is advancing quickly and that compared to datasets collected today, datasets from two years ago are likely to seem smaller and less novel, I ask that the size, novel data

Powered by Editorial Manager® and ProduXion Manager® from Aries Systems Corporation

	types and reuse potential of the dataset described in this manuscript be assessed relative to plant phenomics datasets which are currently published, accessible, and well documented. If we cannot agree on that standard as a community, then when the plant phenomic datasets collected today are ready for publication in 3-5 years, they will face the same challenges of seeming small scale compared to even newer datasets collected with future technology.<br><br>One criteria to review this type of article is dataset size. The authors are claiming a dataset size of ~500Gb, but this includes data (thermal infrared and fluorescence images) that was not mentioned in the manuscript except that it was collected.<br><br>From the terminology used -- the authors are claiming -- I fear the way the manuscript is written somehow gave Prof. Hirsch the impression that I am my co-authors were in bad faith and attempting to deceive readers about the scale of the dataset presented. I would like to state that this was not in any way our intention and I apologize for any ambiguity or apparent deception. We now report the specific percentages of our dataset composed of RGB (51.1%), fluorescence (4.3%), and hyperspectral images (44.6%) prominently when the size of the dataset is first introduced. In addition, as the concern over the lack of analysis from two data types was raised by both reviewers, we had added additional analyses for the fluorescence images to this revised manuscript Thermal IR images previously constituted only 0.4% of the total dataset size, and we have chose to remove this data type from the manuscript to address the concerns of reviewers #1 & #2. - James Schnable.<br><br>I applaud the authors for the willingness to be so open with their data, but I'm not convinced that one month worth of images for 32 genotypes is enough for publication.<br><br>As discussed above, we believe that the unique nature of the hyperspectral images which are part of this dataset, as well as the link to well characterized field phenotyping datasets from the same lines makes this dataset exceptional relative to other plant image datasets.<br><br>2.The manuscripts main point is not to get into conclusions based on their image analysis, but I would have liked to have seen more strenuous ground truthing. The manual measurements were made only at the very last time point. These really should encompass the variation of plants throughout development. How can we determine if the measured traits are accurate at day 9 for example? Nothing can be done for true manual measurements, but digital manual measurements could be made and correlated with image analysis extracted values.<br><br>We thank the reviewer for raising this point. Obviously it is not possible to measure ground truth biomass from digital images, but at the reviewers suggestion we have revisited a sample of images on different days and made manual digital measures of height. The manually counted pixels of plant height is uploaded in Table S1 and this new analysis is presented as Figure 3B.<br><br>In addition, we have added time series ground truth biomass measurements (Table S2) for two genotypes under two drought treatments, including some data previously published as part of Ge et al 2016 doi: 10.1016/j.compag.2016.07.028 and some previously unpublished to demonstrate the correlation between estimated biomass and ground truth biomass at earlier points in the maize lifecycle. These data are currently presented as Figure S1.<br><br>3.Board sense heritability needs to be corrected throughout the manuscript.<br><br>We thank the reviewer for catching this embarrassing repeated spelling error, and we have made corrections throughout the manuscript.
**Additional Information:**	
**Question**	**Response**
Are you submitting this manuscript to a special series or article collection?	No
**Experimental design and statistics**	Yes

Full details of the experimental design and statistical methods used should be given in the Methods section, as detailed in our Minimum Standards Reporting Checklist. Information essential to interpreting the data presented should be made available in the figure legends.<br><br>Have you included all the information requested in your manuscript?	
**Resources**<br><br>A description of all resources used, including antibodies, cell lines, animals and software tools, with enough information to allow them to be uniquely identified, should be included in the Methods section. Authors are strongly encouraged to cite Research Resource Identifiers (RRIDs) for antibodies, model organisms and tools, where possible.<br><br>Have you included the information requested as detailed in our Minimum Standards Reporting Checklist?	Yes
**Availability of data and materials**<br><br>All datasets and code on which the conclusions of the paper rely must be either included in your submission or deposited in publicly available repositories (where available and ethically appropriate), referencing such data using a unique identifier in the references and in the "Availability of Data and Materials" section of your manuscript.<br><br>Have you have met the above requirement as detailed in our Minimum Standards Reporting Checklist?	Yes

Manuscript
Click here to download Manuscript revised_version.pdf

GigaScience, 2017, 1–11

doi: xx.xxxx/xxxx
Manuscript in Preparation
Data Notes

DATA NOTES

Conventional and hyperspectral time-series imaging of maize lines widely used in field trials

Zhikai Liang[1], Piyush Pandey[2], Vincent Stoerger[3], Yuhang Xu[4], Yumou Qiu[4], Yufeng Ge[2] and James C. Schnable[1,*]

[1]Center for Plant Science Innovation, Department of Agronomy and Horticulture, University of Nebraska-Lincoln, Lincoln, 68503, USA and [2]Department of Biological System Engineering, University of Nebraska-Lincoln, Lincoln, 68503, USA and [3]Plant Phenotyping Facilities Manager, University of Nebraska-Lincoln, Lincoln, 68503, USA and [4]Department of Statistics, University of Nebraska-Lincoln, Lincoln, 68503, USA

*schnable@unl.edu

Abstract

Background: Maize (*Zea mays* ssp. *mays*) is one of three crops, along with rice and wheat, responsible for more than 1/2 of all calories consumed around the world. Increasing the yield and stress tolerance of these crops is essential to meet the growing need for food. The cost and speed of plant phenotyping is currently the largest constraint on plant breeding efforts. Datasets linking new types of high throughput phenotyping data collected from plants to the performance of the same genotypes under agronomic conditions across a wide range of environments are essential for developing new statistical approaches and computer vision based tools.
Findings: A set of maize inbreds – primarily recently off patent lines – were phenotyped using a high throughput platform at University of Nebraska-Lincoln. These lines have been previously subjected to high density genotyping, and scored for a core set of 13 phenotypes in field trials across 13 North American states in two years by the Genomes to Fields consortium. A total of 485 GB of image data including RGB, hyperspectral, fluorescence and thermal infrared photos has been released.
Conclusions: Correlations between image-based measurements and manual measurements demonstrated the feasibility of quantifying variation in plant architecture using image data. However, naive approaches to measuring traits such as biomass can introduce nonrandom measurement errors confounded with genotype variation. Analysis of hyperspectral image data demonstrated unique signatures from stem tissue. Integrating heritable phenotypes from high-throughput phenotyping data with field data from different environments can reveal previously unknown factors influencing yield plasticity.

Key words: Maize; Image; Phenomics; Field-phenotype

Data Description

Background

The green revolution created a significant increase in the yields of several major crops in the 1960s and 1970s, dramatically reducing the prevalence of hunger and famine around the world, even as population growth continued. One of the major components of the green revolution was new varieties of major grain crops produced through conventional phenotypic selection with higher yield potentially. Since the green revolution, the need for food has continued to increase, and a great deal of effort in the public and private sectors is devoted to developing crop varieties with higher yield potential. However, as the low hanging fruit for increased yield vanish, each new increase in yield requires more time and resources. Recent studies have

Compiled on: September 21, 2017.
Draft manuscript prepared by the author.

demonstrated that yield increases may have slowed or stopped for some major grain crops in large regions of the world [16]. New approaches to plant breeding must be developed if crop production continues to grow to meet the needs of an increasing population around the world.

The major bottleneck in modern plant breeding is phenotyping. Phenotyping can be used in two ways. Firstly, by phenotyping a large set of lines, a plant breeder can identify those lines with the highest yield potential and/or greatest stress tolerance in a given environment. Secondly, sufficiently detailed phenotyping measurements from enough different plants can be combined with genotypic data to identify regions of the genome of a particular plant species which carry beneficial or deleterious alleles. The breeder can then develop new crop varieties which incorporate as many beneficial alleles and exclude as many deleterious alleles as possible. Phenotyping tends to be expensive and low throughput, yet as breeders seek to identify larger numbers of alleles each with individually smaller effects, the amount of phenotyping required to achieve a given increase in yield potential is growing. High throughput computer vision based approaches to plant phenotyping have the potential to ameliorate this bottleneck. These tools can be used to precisely quantify even subtle traits in plants and will tend to decrease in unit cost with scale, while conventional phenotyping, which remains a human labor intensive processes, does not.

Several recent pilot studies have applied a range of image-processing techniques to extract phenotypic measurements from crop plants. RGB (R: Red channel; G: Green channel; B: Blue channel) camera technology, widely used in the consumer sector, has also been the most widely used tool in these initial efforts at computer vision based plant phenotyping ~~[31, 8, 36]~~[18, 31, 8, 36]. Other types of cameras including fluoresence [5, 4] and NIR (near-infrared) ~~[5, 10]~~ [26, 5, 10] have also been employed in high throughput plant phenotyping efforts, primarily in studies of the response of plant to different abiotic stresses.

However, the utility of current studies is limited in two ways. Firstly, current analysis tools can extract only a small number of different phenotypic measurements from images of crop plants. Approximately 150 tools for analyzing plant image data are listed in a field specific database, however the majority of these are either developed specifically for *Arabidopsis thaliana* which is a model plant, or are designed specifically to analyze images of roots [24]. Secondly, a great deal of image data is generated in controlled environments, however, there are comparatively few attempts to link phenotypic measurements in the greenhouse to performance in the field. However, one recent report in maize suggested that more than 50% of the total variation in yield under field conditions could be predicted using traits measured under controlled environments [36].

Advances in computational tools for extracting phenotypic measurements of plants from image data and statistical models for predicting yield under different field conditions from such measurements requires suitable training datasets. Here, we generate and validate such a dataset consisting of high throughput phenotyping data from 32 distinct maize (*Zea mays*) accessions drawn primarily from recently off-patent lines developed by major plant breeding companies. These accessions were selected specifically because paired data from the same lines exists for a wide range of plant phenotypes collected in 54 distinct field trials at locations spanning 13 North American states or provinces over two years [3]. This extremely broad set of field sites captures much of the environmental variation among areas in which maize are cultivated with total rainfall during the growing season ranging from 133.604 mm to 960.628 mm (excluding sites with supplemental irrigation) and peak temperatures during the growing season ranging from 23.5°C to 34.9°C. In addition, the same lines have been genotyped for approximately 200,000 SNP markers using GBS [3]. Towards these existing data, we added RGB, thermal infra-red, fluorescent and hyperspectral images collected once per day per plant, as well as detailed water-use information (single day, single plant resolution). At the end of the experiment, 12 different types of ground-truth phenotypes were measured for individual plants including destructive measurements. A second experiment focused on interactions between genotype and environmental stress, collecting the same types of data described above from two maize genotypes under well watered and water stressed conditions [13]. We are releasing this curated dataset of high throughput plant phenotyping ~~image~~ images from accessions where data on both genotypic variation and and agronomic performance under field conditions is already available. ~~This~~ **All data was generated using a Lemnatec designed high throughput greenhouse-based phenotyping system constructed at the University of Nebraska-Lincoln. This system is distinguished from existing public sector phenotyping systems in North America by both the ability to grow plants to a height of 2.5 meters and the incorporation of a hyperspectral camera [10]. Given the unique properties described above, this** comprehensive data set should lower the barriers to the development of new computer vision approaches or statistical methodologies by independent researchers who do not have the funding or infrastructure to generate the wide range of different types of data needed.

Methods

Greenhouse Management

All imaged plants were grown in the greenhouse facility of the University of Nebraska-Lincoln's Greenhouse Innovation Center (Latitude: 40.83, Longitude: -96.69) between October 2nd, 2015 to November 10th, 2015. Kernels were sown in 1.5 gallon pots with Fafard germination mix supplemented with 1 cup (236 mL) of Osmocote plus 15-9-12 and one tablespoon (15 mL) of Micromax Micronutrients per 2.8 cubic feet (80 L) of soil. The target photoperiod was 14:10 with supplementary light provided by LED growth lamps from 07:00 to 21:00 each day. The target temperature of the growth facility was between 24 - 26°C. Pots were weighed once per day and watered back to a target weight of 5,400 grams from 10-09-2015 to 11-07-2015 and a target weight of 5,500 grams from 11-08-2015 to the termination of the experiment.

Experimental Design

A total of 156 plants, representing the 32 genotypes listed in Table 1 were grown and imaged, as well as 4 pots with soil but no plant which serve as controls for the amount of water lost from soil as a result of non-transpiration mechanisms (e.g. evaporation). The 156 plants plus control pots were arranged in a ten row by sixteen column grid, with 0.235 meter spacing between plants in the same row and 1.5 meters spacing between rows (Table 2). Sequential pairs of two rows were consisted of a complete replicate with either 31 genotypes and one empty control pot, or 32 genotypes. Within each pair of rows, genotypes were blocked in groups of eight (one half row), with order randomized within blocks between replicates in order to maximize statistical power to analyze within-greenhouse variation.

Plant imaging

The plants were imaged daily using four different cameras in separate imaging chambers. The four types of cameras were thermal infrared, fluorescence, conventional RGB, and hyperspectral [13]. Images were collected in the order that the cam-

Table 1. 32 genotypes in maize phenotype map

Genotype ID	Genotype	Source	Released Year
ZL1	740	Novartis Seeds	1998
ZL2	2369	Cargill	1989
ZL3	A619	Public Sector	1992
ZL4	A632	Public Sector	1992
ZL5	A634	Public Sector	1992
ZL6	B14	Public Sector	1968
ZL7	B37	Public Sector	1971
ZL8	B73	Public Sector	1972
ZL9	C103	Public Sector	1991
ZL10	CM105	Public Sector	1992
ZL11	LH123HT	Holden's Foundation	1984
ZL12	LH145	Holden's Foundation	1983
ZL13	LH162	Holden's Foundation	1990
ZL14	LH195	Holden's Foundation	1989
ZL15	LH198	Holden's Foundation	1991
ZL16	LH74	Holden's Foundation	1983
ZL17	LH82	Holden's Foundation	1985
ZL18	Mo17	Public Sector	1964
ZL19*	DKPB80	DEKALB Genetics	?
ZL20	PH207	Pioneer Hi-Bred	1983
ZL21	PHB47	Pioneer Hi-Bred	1983
ZL22**	PHG35	Pioneer Hi-Bred	1983
ZL23	PHG39	Pioneer Hi-Bred	1983
ZL24	PHG47	Pioneer Hi-Bred	1986
ZL25	PHG83	Pioneer Hi-Bred	1985
ZL26	PHJ40	Pioneer Hi-Bred	1986
ZL27	PHN82	Pioneer Hi-Bred	1989
ZL28	PHV63	Pioneer Hi-Bred	1988
ZL29	PHW52	Pioneer Hi-Bred	1988
ZL30	PHZ51	Pioneer Hi-Bred	1986
ZL31	W117HT	Public Sector	1982
ZL32	Wf9	Public Sector	1991

* Not currently available for order.
** Genotype represented by only a single plant in the dataset.

era types are listed in the previous sentence. On each day, plants were imaged sequentially by row, starting with row 1 column 1 and concluding with row 10, column 16 (Table 2).

~~The thermal infrared camera captured images with a resolution of 480 × 640 pixels and measures emissions at wavelengths between 8–14 µm.~~ Plants were imaged from the side at two angles offset 90 degrees from each other as well as a top down view. On the first day of imaging or when plants reached the two leaf stage of development, the pot was rotated so that the major axis of leaf phylotaxy was parallel to the camera in the PA0 orientation and perpendicular to the camera in the PA90 orientation. This orientation is consistent for all cameras and was not adjusted again for the remainder of the experiment. The fluorescence camera captured images with a resolution of 1038 × 1390 pixels and measures emission intensity at wavelengths between 500-750 nm based on excitation with light at 400-500 nm. Plants were imaged using the same three perspectives employed for the thermal infrared camera. The RGB camera captured images with a resolution of 2454 × 2056 pixels. Initially the zoom of the RGB camera in side views was set such that each pixel corresponds to 0.746 mm at the distance of the pot from the camera. Between 2015-11-05 and 2015-11-10, the zoom level of the RGB camera was reduced to keep the entire plant in the frame of the image. As a result of a system error, this same decreased zoom level was also applied to all RGB images taken on 2015-10-20. At this reduced zoom level, each pixel corresponds to 1.507 mm at the distance of the pot from the camera, an approximate 2x change. Plants were also imaged using the same three perspectives employed for the thermal infrared camera.

The hyperspectral camera captured images with a resolution of 320 horizontal pixels. As a result of the scanning technology employed, vertical resolution ranged from 494 to 499 pixels. Hyperspectral imaging was conducted using illumination from halogen bulbs (Manufacturer Sylvania, model # ES50 HM UK 240V 35W 25° GU10). A total of 243 separate intensity values were captured for each pixel spanning a range of light wavelengths between 546nm-1700nm. Data from each wavelength was stored as a separate grayscale image.

Ground Truth Measurement

Ground truth measurements were collected at the termination of data collection on November 11-12, 2015. Manually collected phenotypes included plant height, total number of visible leaves, number of total fully extended leaves, stem diameter at the base of the plant, stem diameter at the collar of the top fully extended leaf, length and width of top fully extended leaf, and presence/absence visible anthocyanin production in the stem. After these measurements, total above-ground fresh weight biomass was measured for four out of five replicates, resulting in the destruction of the plants. Ground truth data for the drought stressed subset of this dataset was collected following the procedure previously described in [13].

RGB image processing

Pixels covering portions of the plant were segmented out of RGB images using a green index ((2×G)/(R+B)). Pixels with an index value greater than 1.15 [13] were considered to be plant pixels. This method produced some false positive plant pixels within the reflective metal columns at the edge of the image. To reduce the impact of false positives, these areas were excluded from the analysis. Therefore, when plant leaves cross the reflective metal frame, some true plant pixels were excluded. If no plant pixels were identified in the image – often the case in the first several days when the plant had either not germinated or had not risen above the edge of the pot – the value was recorded as "NA" in the output file.

Heritability analysis

A linear regression model was used to analyze the genotype effect (excluding genotype ZL22 which lacked replication) and greenhouse position effect on plant traits. The responses were modeled independently for each day as

$$y_{h,ij,t} = \mu_{h,t} + \alpha_{h,i,t} + \gamma_{h,\nu(i,j),t} + \epsilon_{h,ij,t}, \quad (1)$$

where the subscript $h = 1, \ldots, 6$ denotes the three responses extracted from the images: plant height, width and size for the two views 0 and 90 degree. The subscripts i, j and t denote the jth plant in the ith row and day t, respectively, and $\nu(i,j)$ stands for the genotype at this pot. The parameters α and γ denote row effect and genotype effect, respectively. The error term is $\epsilon_{h,ij,t}$. Let $SS_{\alpha,t}$, $SS_{\gamma,t}$ and $SS_{\epsilon,t}$ be the sum of squares of the regression model (1) for the row effect, genotype effect and the error at time t, respectively. Let $SS_t = SS_{\alpha,t} + SS_{\gamma,t} + SS_{\epsilon,t}$ be the total sum of squares at time t. The heritability HR_t (2) of a given trait within this population was defined as the ratio of the genotype sum of squares over the sum of genotype and error sum of squares. For the estimate of the heritability of measurement error, the row effect term was replaced by a replicate effect (each replicate consisted of two sequential rows) ~~. For this analysis, ZL14, ZL26 were excluded as ground truth measurements were missing for two or more of the five replicated plants, and~~ with exclusion of ZL22 ~~was excluded~~ as only one plant of this genotype was grown.

Table 2. Experimental layout (ID: ZL1-ZL32). At the time this experiment was conducted, the total size of the UNL greenhouse system was ten rows by twenty columns. Positions marked with UP indicate pots filled with plants from an unrelated experiment, while positions marked with NA indicate pots which had no plants. The first complete replicate is shown in color, and the four incomplete blocks within the first replicate are marked in different colors. * marks empty pots within the experimental design.

9	7	3	10	23	25	26	19	13	5	29	21	2	4	18	20	UP	UP	UP	UP
11	16	1	32	17	27	6	22	24	31	14	30	15	28	8	12	UP	UP	UP	UP
29	31	15	13	1	17	25	9	21	30	3	5	*	19	14	6	UP	UP	UP	UP
12	23	32	16	7	28	2	18	10	11	8	26	27	4	20	24	UP	UP	UP	UP
25	9	21	27	28	12	5	11	15	6	*	7	4	23	31	20	UP	UP	UP	UP
19	32	29	24	16	13	3	8	17	14	18	30	10	26	1	2	UP	UP	UP	UP
8	1	17	23	21	5	7	24	27	18	3	11	31	15	19	2	NA	NA	NA	NA
25	30	4	9	16	32	14	20	*	10	6	29	28	12	26	13	NA	NA	NA	NA
15	10	5	32	31	21	16	26	2	18	9	25	6	8	24	*	NA	NA	NA	NA
29	13	23	14	27	7	11	30	12	1	28	4	3	20	17	19	NA	NA	NA	NA

$$\mathrm{HR}_t = \frac{\mathrm{SS}_{\gamma,t}}{\mathrm{SS}_{\epsilon,t} + \mathrm{SS}_{\gamma,t}}. \quad (2)$$

As the heritability index may change over the growth of the plant, an nonparametric smoothing method was provided for analyzing the time varying heritability of plants. The definition in (3) excludes the variation brought by the greenhouse row effect, which can be considered as the percentage of the variation in plant response that can be explained by the genotype effect after adjusting the environmental effect. To compare with this definition of heritability (2), the response in the model without considering the row effect was constructed as

$$y_{h,ij,t} = \mu_{h,t} + \gamma_{h,\nu(i,j),t} + \epsilon_{h,ij,t}, \quad (3)$$

where similarly as (1), $\nu(i,j)$ is the genotype of the jth plant in the ith row. Let $\widetilde{\mathrm{SS}}_{\gamma,t}$ and $\widetilde{\mathrm{SS}}_t$ be the genotype sum of squares and total sum of squares under (4). The classical heritability is defined as

$$\widetilde{\mathrm{HR}}_t = \frac{\widetilde{\mathrm{SS}}_{\gamma,t}}{\widetilde{\mathrm{SS}}_t}. \quad (4)$$

Hyperspectral image processing

Two methods and thresholds were used to extract plant regions of interest from hyperspectral images. First, the commonly used NDVI (normalized difference vegetation index) formula was applied to all pixels using the formula $(R_{750nm} - R_{705nm})/(R_{750nm} + R_{705nm})$, and pixels with a value greater than 0.25 were classified as originating from the plant [12]. Second, based on the difference in reflectance between stem and leaves at wavelengths of 1056nm and 1151nm, the stem was segmented from other part of plants by selecting pixels where (R_{1056nm}/R_{1151nm}) produced a value greater than 1.2. Leaf pixels were defined as pixels identified as plant pixels based on NDVI but not classified as stem pixels. In addition to the biological variation between individual plants, overall intensity variation existed both between different plants imaged on the same day and the same plant on different days as a result of changes in the performance of the lighting used in the hyperspectral imaging chamber. To calibrate each individual image and make the results comparable, a python script (hosted on Github; see code availability section) was used to normalize the intensity values of each plant pixel using data from the non-plant pixels in the same image.

In order to visualize variation across 243 separate wavelength measurements across multiple plant images, we used a PCA (Principal Component Analysis) based approach. After the normalization described above, PCA analysis of intensity values for individual pixels was conducted. PCA values of each individual plant pixel per analyzed plant were translated to intensity values using the formula [x-min(x)]/[max(x)-min(x)]. False color RGB images were constructed with the values for the first principal component stored in the red channel, the second principal component in the green channel and the third principal component stored in the blue channel.

Fluorescence image processing

A consistent area of interest was defined for each zoom level to exclude the pot and non-uniform areas of the imaging chamber backdrop. Within that area, pixels with an intensity value greater than 70 in the red channel were considered to be plant pixels. The aggregate fluorescence intensity was defined as the sum of the red channel intensity values for all pixels classified as plant pixels within the region of interest, and the mean fluorescence intensity as the aggregate fluorescence intensity value divided by the number of plant pixels within the region of interest.

Plant biomass prediction

Two methods were used to predict plant biomass. The first was a single variable model based on the number of zoom level adjusted plant pixels identified in the two RGB side view images on a given day. The second was a multivariate model based upon the sum of plant pixels identified in the two RGB side views, sum of plant pixels identified in the two RGB side views plus the RGB top view, aggregate fluorescence intensity in the two side views, aggregate fluorescence intensity in the two side views plus the top view, number of plant stem pixels identified in the hyperspectral image and number of plant leaf pixels identified in the hyperspectral image. Traits were selected to overlap with those employed by (Chen et al [6]) where possible. This multivariate dataset was used to predict plant biomass using linear modeling as well as MARS, Random Forest and SVM [6]. MARS analysis was performed using the R package earth [25], Random Forest with the R package randomForest [23] and SVM with the R package e1071 [9].

Data Validation and quality control

Validation against ground truth measurements

A total of approximately 500 GB of image data was initially generated by the system during the course of this experiment consisting of RGB images (51.1%), fluorescence images (4.3%), and hyperspectral images (44.6%). A subset of the RGB images within this dataset were previously

analyzed in [7], and were made available for download from http://plantvision.unl.edu/dataset under the terms of the Toronto Agreement. To validate the dataset and ensure plants had been properly tracked through both the automated imaging system and ground truth measurements, a simple script was written to segment images into plant and not-plant pixels (Figure 1). Source codes for all validation analysis are posted online (https://github.com/shanwai1234/Maize_Phenotype_Map).

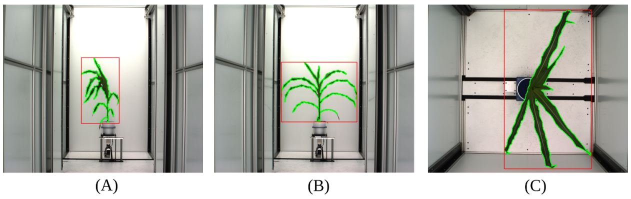

Figure 1. Segmentation of images into plant and not plant pixels for one representative plant (Path to this image in the released dataset: Genotype_ZL019 – > Plant_008-19 – > Image_Type – > Day_32). ~~Area~~ The area enclosed by green border is composed of pixels scored as "plant", the area outside the green border s composed of pixels scored as "not-plant". Minimum bounding rectangle of plant pixels is shown in red. (A) Side view, angle 1; (B) Side view, 90 degree rotation relative to A; (C) Top View.

Based on the segmentation of the image into plant and non-plant pixels, plant height was scored as the y axis dimension of the minimum bounding box. Plant area was scored as the total number of plant pixels observed in both side view images after correcting for the area of each pixel at each zoom employed (See Methods). Similar approaches to estimate plant biomass have been widely employed across a range of grain crop species including rice [1], wheat [15], barley [15, 20], maize [13], sorghum [28] and seteria [10]. Calculated values were compared to manual measurements of plant height and plant fresh biomass which were quantified using destructive methods on the last day of the experiment. ~~In both cases manual measurements and image based measurements were correlated, with calculated height exhibiting a greater correlation with ground truth than biomass (Figure 2A,B).~~ In both cases manual measurements and image derived estimates were highly correlated, although the correlation between manual and estimated height was greater than the correlation between manually measured and estimated biomass (Figure 2A,B). Using the PlantCV software package [14], equivalent correlations between estimated and ground truth biomass were obtained (r=0.91). Estimates of biomass using both software packages were more correlated with each other (r=0.96) than either was with ground truth measurements. This suggests that a significant fraction of the remaining error is the result of the expected imperfect correlation between plant size and plant mass, rather than inaccuracies in easimating plant size using individual software packages. Recent reports have suggested that estimates of biomass incorporating multiple traits extracted from image data can increase accuracy [6]. We tested the accuracy of biomass prediction of four multivariate estimation techniques on this dataset (see Methods). The correlation coefficient (r value) of the estimated biomass measures with ground truth data was 0.949, 0.958, 0.925 and 0.951 for multivariate linear model, MARS, Random Forest and SVM respectively.

~~Plants with ratios of manually measured biomass to plant pixel counts which were distant from the linear regression line (y = 0.617x + 16.702) shown in Figure 2B were individually reexamined. In some cases measurement error was caused by large overlap of leaves at both angles from which the plant had been photographed. However, such obvious cases did not explain the majority of large measurement errors.~~ The residual value – difference between the destructively measured biomass value and the predicted biomass value based on image data and the linear regression line equation – was calculated for each individual plant (Figure 2C). Using data from the multiple replicates of each individual accession, ~~it can be calculated that 62% of the total variation in residual value was controlled by genetic variation between different maize lines~~the proportion of error which is controlled by genetic factors rather than random error can be ascertained. We determined that 58% of the total error in biomass estimate was controlled by genetic variation between different maize lines. As such, this error is systematic rather than random and thus more likely to produce misleading downstream results when used in quantitative genetic analysis. As mentioned above, biomass and plant size are imperfectly correlated, as different plants can exhibit different densities, for example as a result of different leaf to stem ratios. Recent reports have suggested that estimates of biomass incorporating multiple traits extracted from image data can increase accuracy [6]. We tested the accuracy of biomass prediction of four multivariate estimation techniques on this dataset (see Methods). The correlation of the estimated biomass measures with ground truth data was 0.949, 0.958, 0.925 and 0.951 for multivariate linear model, MARS, Random Forest and SVM respectively. However, even when employing the most accurate of these four methods (MARS), 63% of the error in biomass estimation could be explained by genetic factors. This source of error, with the biomass of some lines systematically underestimated and the biomass of other lines systematically overestimated presents a significant challenge to downstream quantitative genetic analysis. Given the prevalence of plant pixel counts as a proxy for biomass [15, 28, 10, 20, 13, 1]~~, this result also highlights the need for more advanced algorithmic approaches to analyze plant image data~~.

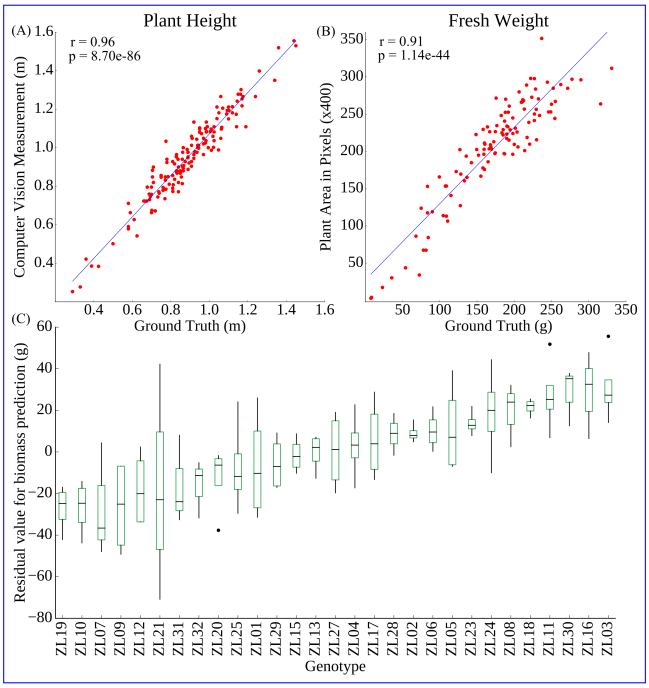

Figure 2. Correlation between image-based and manual measurements of individual plants.(A) Plant height; (B) Plant fresh biomass; (C) Variation in the residual between estimated biomass and ground truth measurement of biomass across inbreds.

Patterns of change over time

One of the desirable aspects of image based plant phenotyping is that, unlike destructively measured phenotypes, the same plant can be imaged repeatedly. Instead of providing a snapshot in time this allows researchers to quantify rates of change in phenotypic values over time, providing an additional set of derived trait values. Given the issues with biomass quantification presented above, measurements of plant height were selected to validate patterns of change in phenotypic values over time. As expected, height increases over time, and the patterns of increase tended to cluster together by genotype (Figure 3A). Increases in height followed by declines, as observed for ZL26, were determined to be caused by a change in the angle of the main stalk. While the accuracy of height estimates was assessed by comparison to physical ground truth measurements only on the last day, the height of three randomly selected plants (Plant 007-26, Plant 002-7 and Plant 041-29) were manually measured from image data and compared to software based height estimates, and no significant differences were observed between the manual and automated measurements (Figure 3B; Supplementary Table 1). To perform a similar test of the accuracy of biomass estimation at different stages in the maize life cycle, a set of existing ground truth measurements for two genotypes under two stress treatments [13] were combined with additional later grow stage data (Supplemental Table 2). The correlation between total plant pixels observed in the two side views and plant biomass was actually substantially higher in this dataset (r=0.97) than the primary dataset, likely as a result of the smaller amount of genetic variability among these plants (Supplementary Figure 1).

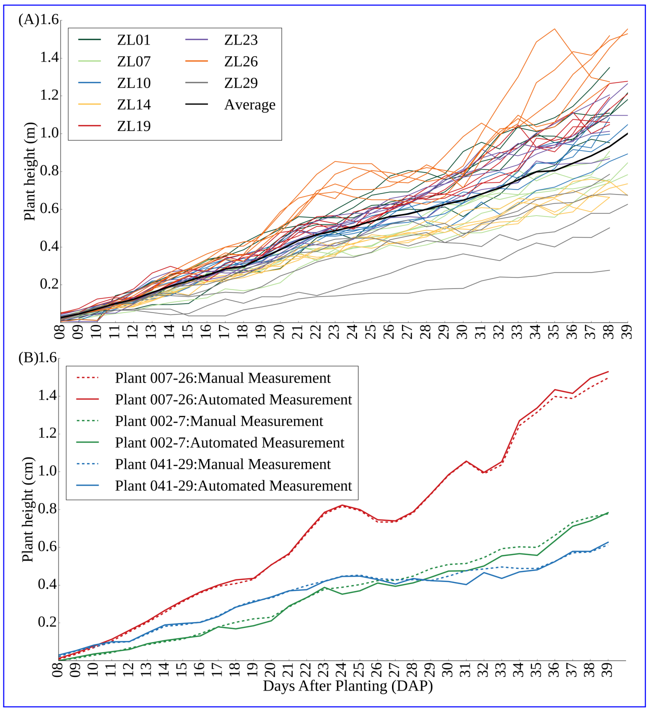

Figure 3. (A) Plant growth curves of each of five replicates of eight selected genotypes; (B) Comparison of manual measurements of plant height from image data with automated measurements for three randomly selected plants on each day of the experiment.

Heritability of phenotypes

The proportion of total phenotypic variation for a trait controlled by genetic variation is referred to as the heritability of that trait and is a good indicator of how easy or difficult it will be to either identify the genes which control variation in a given trait, or to breed new crop varieties in which a given trait is significantly altered. ~~Board-sense~~ Broad-sense heritability can be estimated without the need to first link specific genes to variation in specific traits [19]. Variation in a trait which is not controlled by genotype can result from environmental effects, interactions between genotype and environment, random variance, and measurement error. Controlling for estimated row effects on different phenotypic measurements significantly increased overall broad sense heritability (Figure 4A,B). This result suggests that even within controlled environments such as greenhouses, significant micro-environmental variation exists and that proper statistically based experimental design remains critical importance in even controlled environment phenotyping efforts.

If the absolute size of measurement error was constant in this experiment, as the measured values for a given trait became larger, the total proportion of variation explained by the error term should decrease and, as a result, heritability should increase as observed (Figure 4A). This trend was indeed observed across six different phenotypic measurements (three traits calculated from each of two viewing angles (Figure 4B). Plant height also exhibited significantly greater heritability than plant area or plant width and greater heritability when calculated solely from the 90 degree side angle photo than when calculated solely from to 0 degree angle photo. ~~Plants were initially orientated so that leaves would be arranged parallel to the camera at 0 degrees and perpendicular to the camera at 90 degrees. This initial arrangement was conserved to an extent throughout the experiment, with the 90 degree angle identifying more plant pixels than the 0 degree angle (Figure ??). This difference first becomes significant at 23 DAP (Figure ??), and is consistent with the model described above that if error is constant, larger measured valued will produce higher overall heritability.~~

In previous studies, fluorescence intensity has been treated as an indicator for plant abiotic stress status [32, 11, 4, 21] or chlorophyll content level [27, 17]. Using the fluorescence images collected as part of this experiment, the mean fluorescence intensity value for each plant image was calculated (see Methods). We found that this trait exibited moderate heritability, with the proportion of variation controlled by genetic factors increasing over time and reaching approximately 60% by the last day of the experiment (Figure 4B).

~~Difference in the sum of pixel counts in the two side views of plants on each day of imaging. (A) The median value of total plant pixels across all plants for each angle on each day of the imaging cycle. The difference between the values observed for 0 degree and 90 degree images on each day was tested using a paired t-test. * = P-value < 0.05, ** = P-value < 0.01; (B) 90 degree image of plant 196-19 captured on day 30 (DAP37); (C) 0 degree image of plant 196-19 captured on day 30 (DAP37).~~

Hyperspectral image validation

Hyperspectral imaging of crop plants has been employed previously in field settings using airborne cameras [35, 34, 33]. As a result of the architecture of grain crops such as maize, aerial imagery will largely capture leaf tissue during vegetative growth, and either tassels (maize) or seed heads (sorghum, millet, rice, oats, etc) during reproductive growth. The dataset described here includes hyperspectral imagery taken from the side of individual plants, enabling quantification of the reflectance properties of plant stems in addition to leaf tissue.

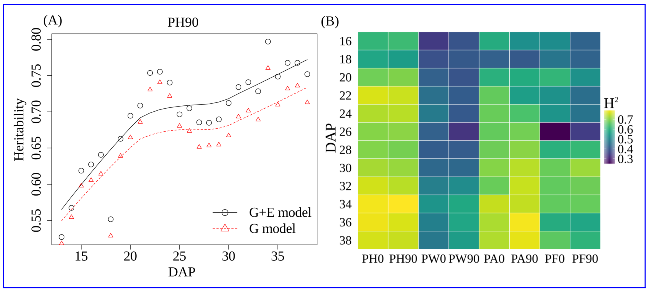

Figure 4. (A) The time course ~~board~~ broad sense heritability of PH90. The heritability in the G model was calculated using a linear model that only considers the effect of genotype with residual values in the error term while heritability in the G + E model was calculated using a linear model that considers the effect of both genotype and environment (row effect) with residual values in the error term.; (B) The time course ~~board~~ broad sense heritability of PA90 before and after controlling for the row effect; (B) Variation in broad-sense heritability (H^2) after controlling row effects for 6 trait measurements every second day across the phenotyping cycle. PA0: Plant Area in 0 degree (The major axis of leaf phylotaxy was parallel to the camera at 0 degree); PA90: Plant Area in 90 degree (The major axis of leaf phylotaxy was perpendicular to the camera at 90 degree); PH0: Plant Height in 0 degree; PH90: Plant Height in 90 degree; PW0: Plant Width in 0 degree; PW90: Plant Width in 90 degree; PF0: Average of plant fluorescence intensity in 0 degree; PF90: Average of plant fluorescence intensity in 90 degree.

Many uses of hyperspectral data reduce the data from a whole plant or whole plot of genetically identical plants to a single aggregate measurement. While these approaches can increase the precision of intensity measurements for individual wavelengths, these approaches also sacrifice spatial resolution and can in some cases produce apparent changes in reflectivity between plants that result from variation in the ratios of the sizes of different organs with different reflective properties. To assess the extent of variation in the reflectance properties of individual plants, a principal component analysis of variation in intensity values for individual pixels was conducted. After non-plant pixels were removed from the hyperspectral data cube (Figure 5A) (See Methods), false color images were generated encoding the intensity values of the first three principal components of variation as the intensity of the red, green, and blue channels respectively (Figure 5B, C and D). The second principal component (green channel) marked boundary pixels where intensity values likely represent a mixture of reflectance data from the plant and from the background. The first principal component (red channel) appeared to indicate distinctions between pixels within the stem of the plant and pixels within the leaves.

Based on this observation, an index was defined which accurately separated plant pixels into leaf and stem (see Methods). Stem pixels were segmented from the rest of the plant using an index value derived from the difference in intensity values observed in the 1056nm and 1151nm hyperspectral bands. This methodology was previously described [13]. The reflectance pattern of individual plant stems is quite dissimilar from the data observed from leaves and exhibits significantly different reflective properties in some areas of the near infrared (Figure 6). Characteristics of the stem are important breeding targets for both agronomic traits (lodging resistance, yield for biomass crops) and value added traits (biofuel conversion potential for bioenergy crops, yield for sugarcane and sweet sorghum). Hyperspectral imaging of the stem has the potential to provide nondestructive measurements of these traits. The calculated pattern of leaf reflectance for the data presented here are comparable with those observed in field-based hyperspectral studies [30, 37, 2], providing both external validation and suggesting that the data presented here may be of use in developing new indices for use under field conditions.

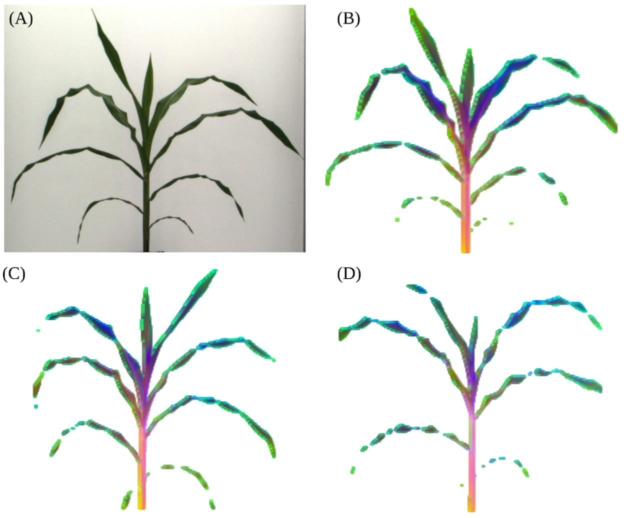

Figure 5. Segmentation and visualization of variation in hyperspectral signatures of representative maize plant images. (A) RGB photo of Plant 013-2 (ZL02) collected on DAP 37; (B) False color image constructed of the same corn plant from a hyperspectral photo taken on the same day. For each plant pixel the values for each of the first three principal components of variation across 243 specific wavelength intensity values are encoded as one of the three color channels in the false image; (C) Equivalent visualization for Plant 048-9 (ZL09); (D) Equivalent visualization for Plant 008-19 (ZL19).

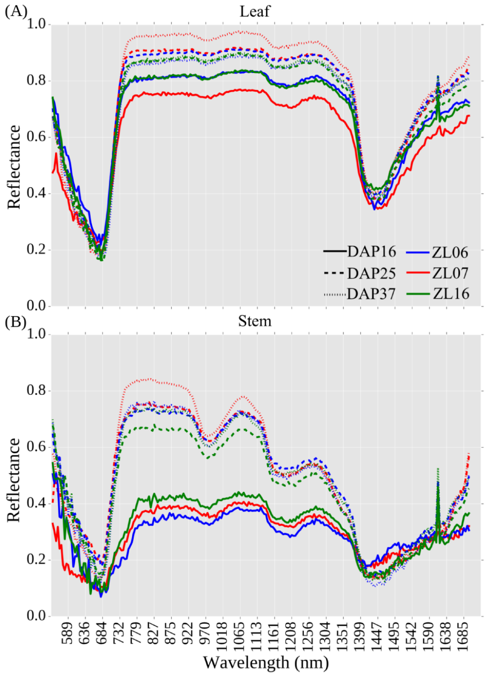

Figure 6. Reflectance values for three plants - Plant 090-6 (ZL06), Plant 002-7 (ZL07), and Plant 145-16 (ZL16) on three days across development. (A) Reflectance values for non-stem plant pixels (~~ie~~ i.e. leaves) (B) Reflectance values for pixels within the plant stem.

In conclusion, while the results presented above highlight some of the simplest traits which can be extracted from plant image data, these represent a small fraction of the total set of phenotypes for which image analysis algorithms currently exist, and those in turn represent a small fraction of the total set of phenotypes which can potentially be scored from image data. Software packages already exist to measure a range of plant architectural traits such as leaf length, angle, and curvature from RGB images [5, 22]. Tools are also being developed to extract phenotypic information on abiotic stress response patterns from fluorescence imaging [5, 4]. The analysis of plant traits from hyperspectral image data, while common place in the remote sensing realm where an entire field may represent a single data point, is just beginning for single plant imaging. Recent work as highlighted the potential of hyperspectral imaging to quantify changes in plant composition and nutrient content throughout development [13, 29]. While these techniques have great potential to accelerate efforts to link genotype to phenotype through ameliorating the current bottleneck of plant phenotypic data collection, it will be important to balance the development of new image analysis tools with the awareness of the potential for systematic error resulting from genetic variation between different lines of the same crop species.

Availability of source code and requirements

- Project name: Maize Phenotype Map
- Project home page: https://github.com/shanwai1234/Maize_Phenotype_Map
- Operating system(s): Linux
- Programming language: Python 2.7
- Other requirements: OpenCV module 2.4.8, Numpy >1.5, CMake > 2.6, GCC > 4.4.x, Scipy 0.13
- License: BSD 3-Clause License

Availability of supporting data and materials

The image data sets, pot weight records per day and ground truth measurements with corresponding documentations from 4 types of cameras of 32 maize inbreds and same types of image data for the two maize genotypes under two stress treatments were deposited in the CyVerse data commons under a CC0 license with doi: 10.7946/P22K7V. (The data for the peer review process can be downloaded from https://doi.org/10.7946/P22K7V). All image data were stored in the following data structure: Genotype – > Plant – > Camera type – > Day. For the hyperspectral camera each photo is stored as 243 sub images, each image representing intensity values for a given wavelength, so these require one additional level of nesting in the data structure Day – > wavelength. The grayscale images from the IR camera and the hyperspectral imaging system are stored as three-channel images with all three channels in a given pixel set to identical values. The fluorescence images contain almost all information in the red channel with the blue and green channel having intensities equal to or very close to zero, but data all three channels exist. Genotype data of 32 inbreds were generated as part of a separate project and can be retrieved from either https://doi.org/10.7946/P2V888 or http://cbsusrv04.tc.cornell.edu/users/panzea/download.aspx?filegroupid=~~4.~~ 4. Measurements for thirteen core phenotypes at each field trial as well as local weather data can be retrieved from publicly released Genomes 2 Fields datasets released on CyVerse. Data from the 2014 G2F field trials is posted (https://doi.org/10.7946/P2V888) and data from the 2015 G2F field trials is posted (https://doi.org/10.7946/P24S31). Genetically identical seeds from the majority of the accessions used in creating both this dataset and the genomes to fields field trial data can be ordered from public domain sources (e.g. USDA GRIN) and are listed in Table 1.

Declarations

List of abbreviations

DAP: Days after planting
GBS: Genotyping by Sequencing
LED: Light-emitting diode
MARS: Multivariate Adaptive Regression Splines
NDVI: Normalized difference vegetation index
NIR: Near-infrared
RGB: ~~Red, Blue and Green~~ An image with separate intensity values for the red, blue and green channels
SNP: Single Nucleotide Polymorphism
SVM: Support Vector Machines
UNL: University of Nebraska-Lincoln
PA0: Plant Area calculated from a 0 degree image. Plants were initially orientated then leaves would be arranged parallel to the camera at 0 degrees.
PA90: Plant Area calculated from a 90 degree image. Plants were initially orientated then leaves would be arranged perpendicular to the camera at 90 degrees.
PCA: Principal Component Analysis
PH0: Plant Height calculated from a 0 degree image
PH90: Plant Height calculated from a 90 degree image
PW0: Plant Width calculated from a 0 degree image
PW90: Plant Width calculated from a 90 degree image
PF0: Average of plant fluorescence intensity in 0 degree
PF90: Average of plant fluorescence intensity in 90 degree.

Consent for publication

Not applicable.

Competing Interests

The authors declare that they have no competing interests.

Funding

This research was supported by the Nebraska Corn Board (Award #88-R-1617-03), the Iowa Corn Board (Award #), the National Science Foundation under Grant No. OIA-1557417, and Internal University of Nebraska funding to JCS. The sources of funding have no role in the design of the study and collection, analysis, and interpretation of data and in writing the manuscript.

Author's Contributions

JCS, and YQ designed the experiment; VS, JCS and ZL performed data acquisition; ZL, PP, YQ, YX, YG and JCS analyzed and interpreted the data; ZL and JCS produced and curated the metadata; ZL and JCS implemented software; ZL and JCS prepared the initial draft. All authors reviewed the manuscript.

Acknowledgements

The authors are grateful to Yang Zhang, Xianjun Lai and Daniel WC Ngu for help in collecting manual measurements of plants, Thomas Hoban for manually counting pixels of selected plant images, Kent M. Eskridge for valuable discussions on experimental design, Addie Thompson, Jinliang Yang for assistance on heritability analysis, and the members of the Genomes to Fields consortium for sharing both seed and datasets prior to publication. CyVerse is supported by the U.S. National Science Foundation under award numbers DBI-0735191 and DBI-1265383.

References

1. Al-Tamimi N, Brien C, Oakey H, Berger B, Saade S, Ho YS, et al. Salinity tolerance loci revealed in rice using high-throughput non-invasive phenotyping. Nature communications 2016;7.
2. Baranowski P, Jedryczka M, Mazurek W, Babula-Skowronska D, Siedliska A, Kaczmarek J. Hyperspectral and thermal imaging of oilseed rape (Brassica napus) response to fungal species of the genus Alternaria. PloS one 2015;10(3):e0122913.
3. Campbell D, de Leon N, Edwards J, Gardiner J, Al Khalifah N, Lawrence-Dill C, et al. Genomes to Fields 2016 data release. CyVerse Data Commons 2016;.
4. Campbell MT, Knecht AC, Berger B, Brien CJ, Wang D, Walia H. Integrating image-based phenomics and association analysis to dissect the genetic architecture of temporal salinity responses in rice. Plant physiology 2015;168(4):1476–1489.
5. Chen D, Neumann K, Friedel S, Kilian B, Chen M, Altmann T, et al. Dissecting the phenotypic components of crop plant growth and drought responses based on high-throughput image analysis. The Plant Cell 2014;26(12):4636–4655.
6. Chen D, Shi R, Pape JM, Klukas C. Predicting plant biomass accumulation from image-derived parameters. bioRxiv 2016;p. 046656.
7. Choudhury SD, Stoerger V, Samal A, Schnable JC, Liang Z, Yu JG. Automated Vegetative Stage Phenotyping Analysis of Maize Plants using Visible Light Images. Data Science for Food, Energy and Water workshop, San Francisco, California, USA, August 2016;.
8. Das A, Schneider H, Burridge J, Ascanio AKM, Wojciechowski T, Topp CN, et al. Digital imaging of root traits (DIRT): a high-throughput computing and collaboration platform for field-based root phenomics. Plant methods 2015;11(1):51.
9. Dimitriadou E, Hornik K, Leisch F, Meyer D, Weingessel A. Misc Functions of the Department of Statistics (e1071), TU Wien. R package version 2005;p. 1–5.
10. Fahlgren N, Feldman M, Gehan MA, Wilson MS, Shyu C, Bryant DW, et al. A versatile phenotyping system and analytics platform reveals diverse temporal responses to water availability in Setaria. Molecular plant 2015;8(10):1520–1535.
11. Fracheboud Y, Haldimann P, Leipner J, Stamp P. Chlorophyll fluorescence as a selection tool for cold tolerance of photosynthesis in maize (Zea mays L.). Journal of experimental botany 1999;50(338):1533–1540.
12. Gamon J, Surfus J. Assessing leaf pigment content and activity with a reflectometer. New Phytologist 1999;143(1):105–117.
13. Ge Y, Bai G, Stoerger V, Schnable JC. Temporal dynamics of maize plant growth, water use, and leaf water content using automated high throughput RGB and hyperspectral imaging. Computers and Electronics in Agriculture 2016;127:625–632.
14. Gehan MA, Fahlgren N, Abbasi A, Berry JC, Callen ST, Chavez L, et al. PlantCV v2. 0: Image analysis software for high-throughput plant phenotyping. PeerJ Preprints; 2017.
15. Golzarian MR, Frick RA, Rajendran K, Berger B, Roy S, Tester M, et al. Accurate inference of shoot biomass from high-throughput images of cereal plants. Plant Methods 2011;7(1):2.
16. Grassini P, Eskridge KM, Cassman KG. Distinguishing between yield advances and yield plateaus in historical crop production trends. Nature communications 2013;4:2918.
17. Guanter L, Zhang Y, Jung M, Joiner J, Voigt M, Berry JA, et al. Global and time-resolved monitoring of crop photosynthesis with chlorophyll fluorescence. Proceedings of the National Academy of Sciences 2014;111(14):E1327–E1333.
18. Hartmann A, Czauderna T, Hoffmann R, Stein N, Schreiber F. HTPheno: an image analysis pipeline for high-throughput plant phenotyping. BMC bioinformatics 2011;12(1):148.
19. Holland JB, Nyquist WE, Cervantes-Martínez CT. Estimating and interpreting heritability for plant breeding: an update. Plant breeding reviews 2003;22:9–112.
20. Honsdorf N, March TJ, Berger B, Tester M, Pillen K. High-throughput phenotyping to detect drought tolerance QTL in wild barley introgression lines. PLoS One 2014;9(5):e97047.
21. Kalaji HM, Jajoo A, Oukarroum A, Brestic M, Zivcak M, Samborska IA, et al. Chlorophyll a fluorescence as a tool to monitor physiological status of plants under abiotic stress conditions. Acta Physiologiae Plantarum 2016;38(4):102.
22. Klukas C, Chen D, Pape JM. Integrated analysis platform: an open-source information system for high-throughput plant phenotyping. Plant physiology 2014;165(2):506–518.
23. Liaw A, Wiener M, et al. Classification and regression by randomForest. R news 2002;2(3):18–22.
24. Lobet G, Draye X, Périlleux C. An online database for plant image analysis software tools. Plant methods 2013;9(1):38.
25. Milborrow S. Earth: multivariate adaptive regression spline models. R package version 2014;3:2–7.
26. Munns R, James RA, Sirault XR, Furbank RT, Jones HG. New phenotyping methods for screening wheat and barley for beneficial responses to water deficit. Journal of Experimental Botany 2010;61(13):3499–3507.
27. Murchie EH, Lawson T. Chlorophyll fluorescence analysis: a guide to good practice and understanding some new applications. Journal of experimental botany 2013;64(13):3983–3998.
28. Neilson EH, Edwards A, Blomstedt C, Berger B, Møller BL, Gleadow R. Utilization of a high-throughput shoot imaging system to examine the dynamic phenotypic responses of a C4 cereal crop plant to nitrogen and water deficiency over time. Journal of experimental botany 2015;66(7):1817–1832.
29. Pandey P, Ge Y, Stoerger V, Schnable JC. High throughput in vivo analysis of plant leaf chemical properties using hyperspectral imaging. Frontiers in Plant Science 2017;8.
30. Smith K, Steven M, Colls J. Use of hyperspectral derivative ratios in the red-edge region to identify plant stress responses to gas leaks. Remote sensing of environment 2004;92(2):207–217.
31. Topp CN, Iyer-Pascuzzi AS, Anderson JT, Lee CR, Zurek PR, Symonova O, et al. 3D phenotyping and quantitative trait locus mapping identify core regions of the rice genome controlling root architecture. Proceedings of the National

Academy of Sciences 2013;110(18):E1695–E1704.

32. Van Kooten O, Snel JF. The use of chlorophyll fluorescence nomenclature in plant stress physiology. Photosynthesis research 1990;25(3):147–150.
33. Yendrek C, Tomaz T, Montes CM, Cao Y, Morse AM, Brown PJ, et al. High-throughput phenotyping of maize leaf physiology and biochemistry using hyperspectral reflectance. Plant physiology 2016;p. pp–01447.
34. Zaman-Allah M, Vergara O, Araus J, Tarekegne A, Magorokosho C, Zarco-Tejada P, et al. Unmanned aerial platform-based multi-spectral imaging for field phenotyping of maize. Plant methods 2015;11(1):35.
35. Zarco-Tejada P, Catalina A, González M, Martín P. Relationships between net photosynthesis and steady-state chlorophyll fluorescence retrieved from airborne hyperspectral imagery. Remote Sensing of Environment 2013;136:247–258.
36. Zhang X, Huang C, Wu D, Qiao F, Li W, Duan L, et al. High-throughput phenotyping and QTL mapping reveals the genetic architecture of maize plant growth. Plant Physiology 2017;p. pp–01516.
37. Zhao D, Reddy KR, Kakani VG, Reddy V. Nitrogen deficiency effects on plant growth, leaf photosynthesis, and hyperspectral reflectance properties of sorghum. European Journal of Agronomy 2005;22(4):391–403.

Supplementary Information

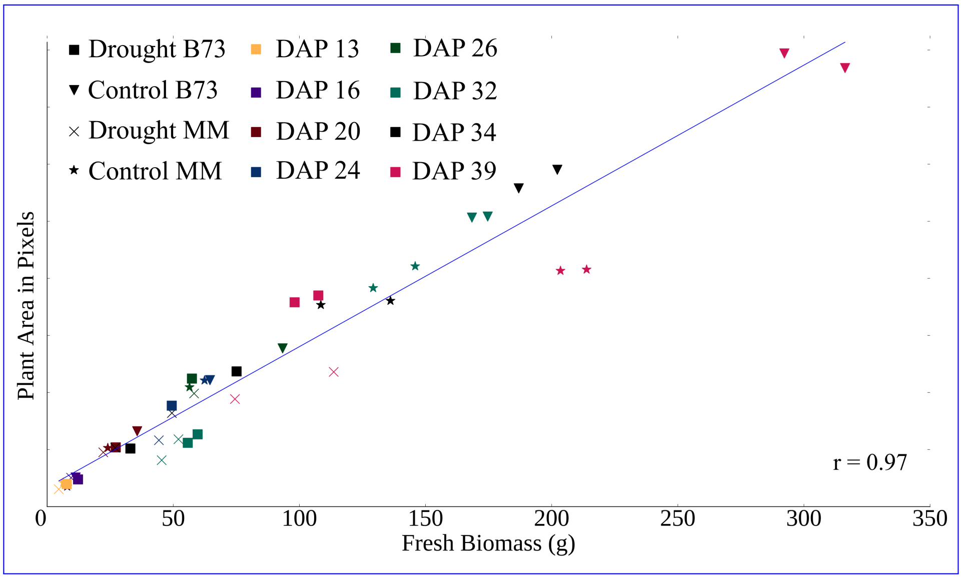

Figure S1. Correlation of fresh weight biomass with total number of plant pixels identified in two side view images for maize plants destructively sampled at eight different time points between 13 days and 39 days after planting (DAP).

Figure S1

Click here to access/download
Supplementary Material
Supplementary Figure 1.png

Table S1

Click here to access/download

Supplementary Material

Supplementary Table 1.xlsx

Table S2

Click here to access/download
Supplementary Material
Supplementary Table 2.xlsx
