# Supplementary figures and images for: Conventional and hyperspectral time-series imaging of maize lines widely used in field trials
Source: Gigascience. 2017 Nov 24;7(2):1–11. doi: 10.1093/gigascience/gix117 (PMC5795349; doi:10.1093/gigascience/gix117)

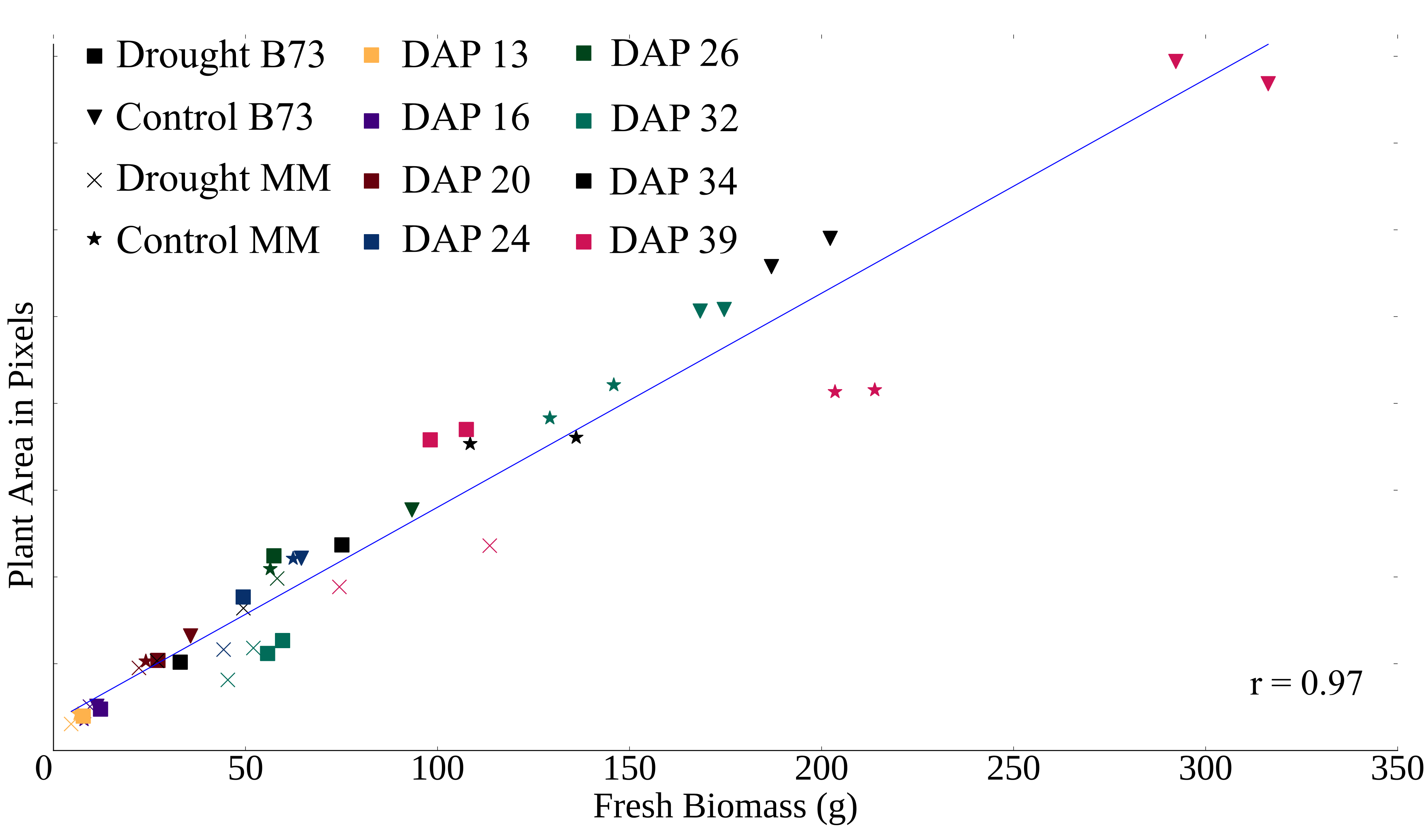

Supplement: Supplement Figure [file gix117_supp.png]
