# Supplementary material for: Conventional and hyperspectral time-series imaging of maize lines widely used in field trials
Source: Gigascience. 2017 Nov 24;7(2):1–11. doi: 10.1093/gigascience/gix117 (PMC5795349; doi:10.1093/gigascience/gix117)
Supplement: Response-to-Reviewer-Comments_Original-Submission.pdf [file gix117_response-to-reviewer-comments_original-submission.pdf]

Author's Response To Reviewer Comments

We would like to thank both the editorial staff of Gigascience and both peer reviewers for taking the time to evaluate the previous draft of this manuscript and provide extremely helpful feedback. It is our belief that this revised manuscript is significantly improved as a result of the changes suggested in the previous round of review.

Reviewer #1:

In the study entitled "Conventional and hyperspectral time-series imaging of maize lines widely used in field trials", Liang et al. performed high-throughput phenotyping (HTP) in a set of maize inbreds. The dataset is especially valuable through integrative analysis with matched field data from different environments and genotypic data.

Major points:
1. It's unclear what automated phenotyping system was used in the study. Is it commercial and available to other users?

This was a serious omission. We have added information on this to the manuscript. This is the first complete dataset to be released from the UNL greenhouse innovation center phenotyping system. The UNL-GIC is based on an Lemnatec/Bosch style design and is available to both internal UNL researchers and external academic and private sector clients. We have added text to the methods and introduction sections to provide the same information summarized in this response.

2. One of the advantages of HTP is to enable us to extract a long list of phenotypic traits for the same plants from image data. However, in the study, the authors only showed some well investigated traits like plant height and projected area. It would be nice to indicate how many potential traits can be extracted from their image data, and how about the potential

We thank the reviewer for this valuable suggestion. We have added an additional concluding paragraph summarizing traits which have been extracted from different image types in the past and discuss other potential phenotypes which it may be possible to extract from these types of images in the future.

3. The authors provided infuorescence and thermal infrared data in the study. But these data were not shown in their analysis, correct? If not, it would be to have an evaluation of these data.

The reviewer is correct that the fluorescence and thermal infrared image data was included in the release but not analyzed in the short results section. Based on the concerns of both reviewer #1 and reviewer #2, in this revised version we include analysis and validation of the fluorescence imaging data -- extracting average fluorescence intensity measurements from plant pixels and testing the heritability of these values -- in results and in figure 4B and the additional code used to perform these analysis has been added to the associated github repository. We have also added discussion and references to previous literature demonstrating the utility of measurements of fluorescence intensity and its correlation to other plant traits such as chlorophyll content and

abiotic stress responses.

4. In figure 2A and 2B, plant height showed higher correlation with manual data but biomass not. This is true because biomass is a complex phenotypic that cannot solely explained by a single measurements (plant project area). I have three related questions here.

1) which project area used for the analysis? PA0 or PA90?

We apologize for the ambiguity. We actually used the sum the the estimated number of plant pixels from the two offset side views. The manuscript has been revised to explicitly state this point at the place where we first introduce biomass estimation.

2) the correlation coefficient (r=0.72) is lower than existing results in maize (e.g., Muraya et al, 10.1111/tpj.13390). Is this possible due to the software used to derive the area measurement? If I correctly read the manuscript, the author used their in-house image processing pipeline. Did the author compare their results to the results generated by some published image-processing software?

We thank the reviewer for this suggestion. We have reestimated plant biomass using PlantCV2 (Noah et al. 2015 DOI: 10.1016/j.molp.2015.06.005; Gehan MA et al. DOI: 10.7287/peerj.preprints.3225v1). The correlation between the sum of plant pixel areas in the two side views and ground truth biomass measurements was (r=0.91). The large increase in accuracy in the PlantCV2 based analysis lead us to reevaluate the approach used in our own scripts, and identify a significant coding flaw which lead to incorrect plant pixel count data being using in our own calculations. We have revised figures 2B, 2C and 4B to use the corrected data, and provide a comparison of biomass estimates from the code used here, PlantCV2's code, and ground truth measurements in the revised manuscript.

3) Given the biomass is not well predicted by a single image-derived parameters, I would suggest to predict it by using multiple parameters (e.g., doi.org/10.1101/046656).

We agree with the reviewer that pixel counts should not have a perfect linear relationship with plant biomass. Factors such as the ratio of stems to leaves, and stem density and diameter vary significantly among genotypes and can produce significant differences in plant pixel counts from plants with equivalent quantities of biomass. We included the analysis of the relationship between plant pixel number and estimated biomass as single variable linear models of the relationship between plant pixel count and biomass are widely used in the literature (Noah F et al. DOI:10.1016/j.molp.2015.06.005; Golzarian MR et al. DOI:10.1186/1746-4811-7-2; Malachy T. Campbell MT et al. DOI: 10.1104/pp.15.00450; Ge Y et al. DOI: 10.1016/j.compag.2016.07.028), and to illustrate there that there is systematic genetically-linked bias in estimates of plant biomass generated using this method.

However, based on this suggestion by the review we now include the results from a second set biomass estimates generated using the four approaches proposed in the manuscript referenced above (multivariate linear model, MARS, Random Forest and SVM).* These models increased the accuracy of plant biomass estimates from r = 0.91 (single variable linear model) to r = 0.96

(the machine learning methods MARS). However, we show than even when using machine learning methods trained to estimate biomass from multiple traits extracted from multiple image types error in biomass estimate remains non-random and systematically biased towards over or under estimation of the biomass of specific genotypes. This information is now included in the main text.

5. Since the plants were randomly set on the pot, in principle there is no difference for the measurement of PA0 and PA90. But they are significantly different (figure 5)?

We appreciate the reviewer raising this point. On the first day of imaging or when plants reach to the two leaf stage, plants were arranged so that the major axis of leaf phylotaxy was parallel to the camera in the PA0 orientation and perpendicular to the camera in the PA90 angle. We have added this information to the manuscript in both the declarations and the legend of figure 4 (where the terms PA0 and PA90 are first introduced). As we feel that this point was a distraction from the central point of the manuscript, we have removed Figure 5 in this revised version of the manuscript.

Reviewer #2: This manuscript describes the generation of a time-series dataset of conventional and hyperspectral images of commonly known and important maize lines. The authors describe the methods of data collection and how it is useful, especially in conjunction with other already available datasets for the same lines. The authors begin to analyze the dataset generated, focusing on biomass measures and determining heritability. The authors conclude that they believe it is important and necessary to combine controlled environment data with field data to tackle problems facing crop production. I do have several comments about the manuscript in its current form:

1. My main concern about the manuscript is the amount of data use in the article. The manuscript was submitted as a 'Data Note', but it is not obvious this data is exceptional, rare, or novel as it was collected nearly 2 years ago.

The reviewer raises two distinct points here. The first is that this dataset may not be exceptional or rare. The second is that this dataset is almost two years old. Of the various points raised by reviewer #2, these concerns appears to be the most critical barriers to publication, so we have responded to them at some length.

Exceptionality of the dataset.

I think the reason the two of us are in disagreement about the exceptionality of this dataset is that we’re comparing this dataset to different baselines. I believe that compared to published plant image datasets, our dataset is exceptional for two reasons. 1) The link between publically available field based data in a wide range of well characterized environments. 2) Hyperspectral imagery collected under controlled light conditions from individual corn (maize) plants.

Now at the same time, because Gigascience practices open peer review, I can see that the reviewer is part of a research group which has done, and continues to do groundbreaking work in both the image collection and phenotypic data extraction portions of plant phenotyping. It

wouldn't surprise me at all if, compared to the internal datasets he and his collaborators have access to, the dataset we are trying to release to the community is rather small and not particularly novel or exciting.

The disconnect between these two assessments is a result of the second point that the reviewer raises.

Age of the dataset.

It has been my observation that the cycle time from data collection to publication in plant phenomics is significantly longer than in other scientific fields in which I have experience (particularly plant comparative genomics where it is often possible to go from idea to analysis to submitted paper in as little as six months). I don't want to base my position on a qualitative gut feeling, so I dug into the literate a bit to see if that gut feeling is supported by quantitative data. In many cases manuscripts do not report the date of phenotypic data collection -- which is whole concern of its own but beside the point today -- but in the cases where dates of data collection were reported, the lag time was quite significant.

Chen et al Dec 2014 10.1105/tpc.114.129601 used data collected in July 2011 (3.5 years)
Campbell et al Aug 2015 10.1104/pp.15.00450 used data collected in 2013 (2.5 years)
Muraya et al Feb 2017 10.1111/tpj.13390 used data collected in 2011-2012 (5+ years)
Zhang et al Mar 2017 10.1104/pp.16.01516 used data collected in 2011-2012 (5+ years)
Feldman et al June 2017 10.1371/journal.pgen.1006841 used data collected in 2013-2014 (3+ years)

I suspect that part of the delay between data collection and publication for plant phenomic datasets is the fact that computational and statistical methods for the analysis of high throughput plant phenotyping data remain much less mature than those for other data types encountered by plants scientists. Right now it is very hard to interest third party computer scientists and statisticians without pre-existing collaborations with plant scientists in developing new methods for plant phenomics given the dearth of well documented and freely usable datasets. It was and is our hope that this dataset and others like it can help to bring more such researchers into the community.

Summary

In conclusion, while I agree with the reviewer that the field of plant phenomics is advancing quickly and that compared to datasets collected today, datasets from two years ago are likely to seem smaller and less novel, I ask that the size, novel data types and reuse potential of the dataset described in this manuscript be assessed relative to plant phenomics datasets which are currently published, accessible, and well documented. If we cannot agree on that standard as a community, then when the plant phenomic datasets collected today are ready for publication in 3-5 years, they will face the same challenges of seeming small scale compared to even newer datasets collected with future technology.

One criteria to review this type of article is dataset size. The authors are claiming a dataset size

of ~500Gb, but this includes data (thermal infrared and fluorescence images) that was not mentioned in the manuscript except that it was collected.

From the terminology used -- the authors are claiming -- I fear the way the manuscript is written somehow gave Prof. Hirsch the impression that I am my co-authors were in bad faith and attempting to deceive readers about the scale of the dataset presented. I would like to state that this was not in any way our intention and I apologize for any ambiguity or apparent deception. We now report the specific percentages of our dataset composed of RGB (51.1%), fluorescence (4.3%), and hyperspectral images (44.6%) prominently when the size of the dataset is first introduced. In addition, as the concern over the lack of analysis from two data types was raised by both reviewers, we had added additional analyses for the fluorescence images to this revised manuscript Thermal IR images previously constituted only 0.4% of the total dataset size, and we have chose to remove this data type from the manuscript to address the concerns of reviewers #1 & #2. - James Schnable.

I applaud the authors for the willingness to be so open with their data, but I'm not convinced that one month worth of images for 32 genotypes is enough for publication.

As discussed above, we believe that the unique nature of the hyperspectral images which are part of this dataset, as well as the link to well characterized field phenotyping datasets from the same lines makes this dataset exceptional relative to other plant image datasets.

2. The manuscripts main point is not to get into conclusions based on their image analysis, but I would have liked to have seen more strenuous ground truthing. The manual measurements were made only at the very last time point. These really should encompass the variation of plants throughout development. How can we determine if the measured traits are accurate at day 9 for example? Nothing can be done for true manual measurements, but digital manual measurements could be made and correlated with image analysis extracted values.

We thank the reviewer for raising this point. Obviously it is not possible to measure ground truth biomass from digital images, but at the reviewers suggestion we have revisited a sample of images on different days and made manual digital measures of height. The manually counted pixels of plant height is uploaded in Table S1 and this new analysis is presented as Figure 3B.

In addition, we have added time series ground truth biomass measurements (Table S2) for two genotypes under two drought treatments, including some data previously published as part of Ge et al 2016 doi: 10.1016/j.compag.2016.07.028 and some previously unpublished to demonstrate the correlation between estimated biomass and ground truth biomass at earlier points in the maize lifecycle. These data are currently presented as Figure S1.

3. Board sense heritability needs to be corrected throughout the manuscript.

We thank the reviewer for catching this embarrassing repeated spelling error, and we have made corrections throughout the manuscript.
