# Supplementary material for: Conventional and hyperspectral time-series imaging of maize lines widely used in field trials
Source: Gigascience. 2017 Nov 24;7(2):1–11. doi: 10.1093/gigascience/gix117 (PMC5795349; doi:10.1093/gigascience/gix117)
Supplement: Response-to-Reviewer-Comments_Revision-1.pdf [file gix117_response-to-reviewer-comments_revision-1.pdf]

Author's Response To Reviewer Comments

I am pleased to inform you that it is potentially acceptable for publication in GigaScience, once you have carried out some final minor essential revisions.

1) Please see the attached PDF with marked up comments which need to be addressed.- note URLs must be moved to the references and only the reference number cited in the main text.

Response: We have moved each URL to references rather than embedding them in text.

2) Please fix this URL which is currently broken:
http://cbsusrv04.tc.cornell.edu/users/panzea/download.aspx?%1Flegroupid=4

Response: We have replaced this link with one to a more general portion of the website which is less likely to stop working, and provided a reference ID readers can use to find the specific dataset we downloaded.

3) once you have made our marked up final edits, please submit a clean version online. Note that we will need all Latex source files PLUS a Word.docx version of the manuscript.

Response: We have made the edited requested in the marked up PDF. We are uploading all LaTeX source files, plus a docx file which we have reviewed to be as close to the original LaTeX as possible (but in the case of any discrepancy between the two, please treat the LaTeX as the ground truth). Note that we had to make one edit to the bst file provided in the GigaScience on Overleaf, as the default bst file was numbering references alphabetically by author's last name, and looking at other papers published in GigaScience it appears the correct style is to number references based on their order of appearance in the manuscript.

Reviewer reports:
Reviewer #2: This manuscript describes the generation of a time-series dataset of conventional and hyperspectral images of commonly known and important maize lines. The authors describe the methods of data collection and how it is useful, especially in conjunction with other already available datasets for the same lines. The authors begin to analyze the dataset generated, focusing on biomass measures and determining heritability. The authors conclude that they believe it is important and necessary to combine controlled environment data with field data to tackle problems facing crop production.

Comments:
I want to clarify my first review of this manuscript. It was not my intention to make it seem as the dataset generated for this manuscript is not important, large, or useful for the broader maize and plant phenotyping community. This dataset could be very useful for some research groups, including the corresponding authors group. The authors response to the age question of the dataset of, look at the cycle time of data collection to publication in plant phenomics is generally longer, I totally agree with. The authors give numerous examples to back up this point. I'm not disputing this, but the authors should also note the amount of downstream analyses and new biological findings that are in these manuscripts as well. The importance of the presented dataset

as outlined by the authors is its ability to link with other already available datasets, which isn't shown in the manuscript. This paper is a data release paper with a valuable, controlled, and well documented dataset. The real value in the dataset will be shown in subsequent publications that begin to combine the multiple datasets available from these maize lines (field phenotyping, genotyping, controlled environment phenotyping).

Response: We are in complete agreement with the reviewer that the real scientific value of this data release will come from downstream analyses and biological insights conducted using the dataset and other other associated datasets from the same maize lines. This manuscript does not include significant new biological findings of our own, it is only an attempt to get our raw data out in a form that other researchers can also use it without waiting the additional 1-3 years it would take (if the timelines of prior plant phenotypic papers are a useful guide) for us to tell our own biological story with this dataset.
