# Supplementary material for: Conventional and hyperspectral time-series imaging of maize lines widely used in field trials
Source: Gigascience. 2017 Nov 24;7(2):1–11. doi: 10.1093/gigascience/gix117 (PMC5795349; doi:10.1093/gigascience/gix117)
Supplement: Reviewer-1-Report-(Original-Submission) -- Dijun Chen — 20 Aug 2017 Reviewed [file gix117_reviewer-1-report-(original-submission).pdf]

Reviewer Report

Title: Conventional and hyperspectral time-series imaging of maize lines widely used in field trials

Version: Original Submission **Date:** 8/20/2017

Reviewer name: Dijun Chen

Reviewer Comments to Author:

In the study entitled "Conventional and hyperspectral time-series imaging of maize lines widely used in field trials", Liang et al. performed high-throughput phenotyping (HTP) in a set of maize inbreds. The dataset is especially valuable through integrative analysis with matched field data from different environments and genotypic data.Major points:1. It's unclear what automated phenotyping system was used in the study. Is it commercial and available to other users?2. One of the advantages of HTP is to enable us to extract a long list of phenotypic traits for the same plants from image data. However, in the study, the authors only showed some well investigated traits like plant height and projected area. It would be nice to indicate how many potential traits can be extracted from their image data, and how about the potential3. The authors provided infuorescence and thermal infrared data in the study. But these data were not shown in their analysis, correct? If not, it would be to have an evaluation of these data.4. In figure 2A and 2B, plant height showed higher correlation with manual data but biomass not. This is true because biomass is a complex phenotypic that cannot solely explained by a single measurements (plant project area). I have three related questions here. 1) which project area used for the analysis? PA0 or PA90? 2) the correlation coefficient (r=0.72) is lower than existing results in maize (e.g., Muraya et al, 10.1111/tpj.13390). Is this possible due to the software used to derive the area measurement? If I correctly read the manuscript, the author used their in-house image processing pipeline. Did the author compare their results to the results generated by some published image-processing software? 3) Given the biomass is not well predicted by a single image-derived parameters, I would suggest to predict it by using multiple parameters (e.g., doi.org/10.1101/046656).5. Since the plants were randomly set on the pot, in principle there is no difference for the measurement of PA0 and PA90. But they are significantly different (figure 5)?

Level of Interest

Please indicate how interesting you found the manuscript: An article whose findings are important to those with closely related research interests

Quality of Written English

Please indicate the quality of language in the manuscript: Acceptable

Declaration of Competing Interests

Please complete a declaration of competing interests, considering the following questions:

- Have you in the past five years received reimbursements, fees, funding, or salary from an organisation that may in any way gain or lose financially from the publication of this manuscript, either now or in the future?
- Do you hold any stocks or shares in an organisation that may in any way gain or lose financially from the publication of this manuscript, either now or in the future?
- Do you hold or are you currently applying for any patents relating to the content of the manuscript?
- Have you received reimbursements, fees, funding, or salary from an organization that holds or has applied for patents relating to the content of the manuscript?
- Do you have any other financial competing interests?
- Do you have any non-financial competing interests in relation to this paper?

If you can answer no to all of the above, write 'I declare that I have no competing interests' below. If your reply is yes to any, please give details below.

I declare that I have no competing interests

I agree to the open peer review policy of the journal. I understand that my name will be included on my report to the authors and, if the manuscript is accepted for publication, my named report including any attachments I upload will be posted on the website along with the authors' responses. I agree for my report to be made available under an Open Access Creative Commons CC-BY license (http://creativecommons.org/licenses/by/4.0/). I understand that any comments which I do not wish to be included in my named report can be included as confidential comments to the editors, which will not be published.

I agree to the open peer review policy of the journal
