# Supplementary material for: Conventional and hyperspectral time-series imaging of maize lines widely used in field trials
Source: Gigascience. 2017 Nov 24;7(2):1–11. doi: 10.1093/gigascience/gix117 (PMC5795349; doi:10.1093/gigascience/gix117)
Supplement: Reviewer-2-Report-(Original-Submission) -- Cory Hirsh — 24 Aug 2017 Reviewed [file gix117_reviewer-2-report-(original-submission).pdf]

Reviewer Report

Title: Conventional and hyperspectral time-series imaging of maize lines widely used in field trials

Version: Original Submission **Date:** 8/24/2017

Reviewer name: Cory Hirsh

Reviewer Comments to Author:

This manuscript describes the generation of a time-series dataset of conventional and hyperspectral images of commonly known and important maize lines. The authors describe the methods of data collection and how it is useful, especially in conjunction with other already available datasets for the same lines. The authors begin to analyze the dataset generated, focusing on biomass measures and determining heritability. The authors conclude that they believe it is important and necessary to combine controlled environment data with field data to tackle problems facing crop production. I do have several comments about the manuscript in its current form:1. My main concern about the manuscript is the amount of data use in the article. The manuscript was submitted as a 'Data Note', but it is not obvious this data is exceptional, rare, or novel as it was collected nearly 2 years ago. One criteria to review this type of article is dataset size. The authors are claiming a dataset size of ~500Gb, but this includes data (thermal infrared and fluorescence images) that was not mentioned in the manuscript except that it was collected. I applaud the authors for the willingness to be so open with their data, but I'm not convinced that one month worth of images for 32 genotypes is enough for publication.2. The manuscripts main point is not to get into conclusions based on their image analysis, but I would have liked to have seen more strenuous ground truthing. The manual measurements were made only at the very last time point. These really should encompass the variation of plants throughout development. How can we determine if the measured traits are accurate at day 9 for example? Nothing can be done for true manual measurements, but digital manual measurements could be made and correlated with image analysis extracted values.3. Board sense heritability needs to be corrected throughout the manuscript.

Level of Interest

Please indicate how interesting you found the manuscript: An article of limited interest

Quality of Written English

Please indicate the quality of language in the manuscript: Acceptable

Declaration of Competing Interests

Please complete a declaration of competing interests, considering the following questions:

- Have you in the past five years received reimbursements, fees, funding, or salary from an organisation that may in any way gain or lose financially from the publication of this manuscript, either now or in the future?
- Do you hold any stocks or shares in an organisation that may in any way gain or lose financially from the publication of this manuscript, either now or in the future?
- Do you hold or are you currently applying for any patents relating to the content of the manuscript?
- Have you received reimbursements, fees, funding, or salary from an organization that holds or has applied for patents relating to the content of the manuscript?
- Do you have any other financial competing interests?
- Do you have any non-financial competing interests in relation to this paper?

If you can answer no to all of the above, write 'I declare that I have no competing interests' below. If your reply is yes to any, please give details below.

I declare that I have no competing interests

I agree to the open peer review policy of the journal. I understand that my name will be included on my report to the authors and, if the manuscript is accepted for publication, my named report including any attachments I upload will be posted on the website along with the authors' responses. I agree for my report to be made available under an Open Access Creative Commons CC-BY license (http://creativecommons.org/licenses/by/4.0/). I understand that any comments which I do not wish to be included in my named report can be included as confidential comments to the editors, which will not be published.

I agree to the open peer review policy of the journal
