# Supplementary material for: Conventional and hyperspectral time-series imaging of maize lines widely used in field trials
Source: Gigascience. 2017 Nov 24;7(2):1–11. doi: 10.1093/gigascience/gix117 (PMC5795349; doi:10.1093/gigascience/gix117)
Supplement: Reviewer-2-Report-(Revision-1) -- Cory Hirsh — 13 Oct 2017 Reviewed [file gix117_reviewer-2-report-(revision-1).pdf]

Reviewer Report

Title: Conventional and hyperspectral time-series imaging of maize lines widely used in field trials

Version: Revision 1 **Date:** 10/13/2017

Reviewer name: Cory Hirsh

Reviewer Comments to Author:

This manuscript describes the generation of a time-series dataset of conventional and hyperspectral images of commonly known and important maize lines. The authors describe the methods of data collection and how it is useful, especially in conjunction with other already available datasets for the same lines. The authors begin to analyze the dataset generated, focusing on biomass measures and determining heritability. The authors conclude that they believe it is important and necessary to combine controlled environment data with field data to tackle problems facing crop production.Comments:I want to clarify my first review of this manuscript. It was not my intention to make it seem as the dataset generated for this manuscript is not important, large, or useful for the broader maize and plant phenotyping community. This dataset could be very useful for some research groups, including the corresponding authors group. The authors response to the age question of the dataset of, look at the cycle time of data collection to publication in plant phenomics is generally longer, I totally agree with. The authors give numerous examples to back up this point. I'm not disputing this, but the authors should also note the amount of downstream analyses and new biological findings that are in these manuscripts as well. The importance of the presented dataset as outlined by the authors is its ability to link with other already available datasets, which isn't shown in the manuscript. This paper is a data release paper with a valuable, controlled, and well documented dataset. The real value in the dataset will be shown in subsequent publications that begin to combine the multiple datasets available from these maize lines (field phenotyping, genotyping, controlled environment phenotyping).

Level of Interest

Please indicate how interesting you found the manuscript: An article of importance in its field

Quality of Written English

Please indicate the quality of language in the manuscript: Acceptable

Declaration of Competing Interests

Please complete a declaration of competing interests, considering the following questions:

- Have you in the past five years received reimbursements, fees, funding, or salary from an organisation that may in any way gain or lose financially from the publication of this manuscript, either now or in the future?

- Do you hold any stocks or shares in an organisation that may in any way gain or lose financially from the publication of this manuscript, either now or in the future?
- Do you hold or are you currently applying for any patents relating to the content of the manuscript?
- Have you received reimbursements, fees, funding, or salary from an organization that holds or has applied for patents relating to the content of the manuscript?
- Do you have any other financial competing interests?
- Do you have any non-financial competing interests in relation to this paper?

If you can answer no to all of the above, write 'I declare that I have no competing interests' below. If your reply is yes to any, please give details below.

I declare that I have no competing interests

I agree to the open peer review policy of the journal. I understand that my name will be included on my report to the authors and, if the manuscript is accepted for publication, my named report including any attachments I upload will be posted on the website along with the authors' responses. I agree for my report to be made available under an Open Access Creative Commons CC-BY license (http://creativecommons.org/licenses/by/4.0/). I understand that any comments which I do not wish to be included in my named report can be included as confidential comments to the editors, which will not be published.

I agree to the open peer review policy of the journal
